# Nitrogen dioxide gas-sensing properties of hydrothermally synthesized WO_3_ · nH_2_O nanostructures

**DOI:** 10.1098/rsos.221135

**Published:** 2023-04-12

**Authors:** Shraddha Hambir, Shweta Jagtap

**Affiliations:** ^1^ Department of Electronic and Instrumentation Science, Savitribai Phule Pune University, Pune 411007, India; ^2^ Department of Physics, Savitribai Phule Pune University, Pune 411007, India

**Keywords:** NO_2_, behaviour, hydrothermally, synthesized, WO_3_, nanostructures

## Abstract

Nitrogen dioxide (NO_2_) has been identified as a serious air pollutant that threats to our environment, human life and world ecosystems. Therefore, detection of this air pollutant is crucial. Metal oxide semiconductor is one of the best approaches frequently used to detect NO_2_ at relatively low temperatures. Hydrated tungsten trioxide (WO_3_ · H_2_O), an n-type semiconductor, is regarded to be a promising material for fabricating gas sensors, which are widely used in environmental and safety monitoring. In this work, WO_3_ · nH_2_O nanoparticles have been synthesized using a polyfunctional surfactant-mediated hydrothermal approach in the addition of H_2_C_2_O_4_ and K_2_SO_4_ at a molar ratio of 1 : 1. This paper has also reported the effect of reaction temperature (120°C to 200°C) on morphological changes and gas-sensing performance. The characterization of these synthesized nanostructures was carried out by UV–Vis absorption spectroscopy, X-ray diffraction and field-emission scanning electron microscopy (FESEM). The UV absorption peak was obtained around 300 nm. FESEM analysis showed sheet-like structures come together to form flower-type morphology. The synthesized WO_3_ · nH_2_O flower-like structures was then used for NO_2_ gas-sensing application. The prepared sensors showed considerably better sensor response (*R*_g_/*R*_a_ = 17.48) at 185°C for 25 ppm NO_2_.

## 1. Introduction

Nitrogen dioxide (NO_2_) gas is one of the main air pollutants that contributes to respiratory infections, acid rain and depletion of ozone layer [1,2]. NO_2_ is emitted by the combustion of fossil fuels and vehicle exhaust that not only causes serious respiratory issues in humans but it can also produce a variety of noxious gases [3,4]. According to an alert from the National Institute for Occupational Safety and Health (NIOSH), NO_2_ can cause death when present at concentrations more than 20 parts per million. Thus, it is necessary to fabricate a high-performance gas sensor which can rapidly, precisely and reliably detect low concentration of NO_2_ in the air. Several types of gas sensors (electrochemical, optical and resistive) have been extensively applied for real-time NO_2_ sensing [5–10]. However, gas sensors based on metal oxide semiconductors (MOSs) have low production costs as well as high sensitivity and selectivity towards desired gases and hence gained a lot of interest in environmental pollution monitoring [11]. Certain semiconductor oxides, including SnO_2_, ZrO_2_, ZnO, CeO_2_, In_2_O_3_, TiO_2_ and WO_3_, are commonly used for their high sensitivity towards specific gases and their ease of manufacturing [12–15]. Especially, tungsten oxide (WO_3_) and its hydrates (WO_3_ · nH_2_O) are one of the most effective n-type MOSs that has been discovered as a potential material for NO_2_ gas monitoring [16]. In the case of gas sensors, morphology, particle size and solubility rate of nanomaterials are all important factors. Therefore, the well-controlled morphology of WO_3_ · nH_2_O needs to be evaluated first, and based on this, research was focused on study of several forms of WO_3_ · xH_2_O such as nanowires, hollow sphere, square-like micro- and nanostructures, nanoparticles, and nanorods [17–20]. This morphology could effectively increase the surface for gas adsorption and desorption, leading to improvement in the gas-sensing performance. To date, several methods such as chemical vapour deposition [21], spray pyrolysis [22], sol–gel [23] sputtering [24], hydrothermal [25], solvothermal approach [26] and other techniques were used to synthesize WO_3_ nanoparticles. The hydrothermal technique is one of the important and commonly used methods for manufacturing of nanoscale materials due to its low-temperature range, easy process management etc. Additionally, it is possible to achieve the improved crystallinity without heat treatment [27,28]. A hydrothermal method is also used to develop free-standing nanostructures with a number of morphologies at low temperatures. These nanostructured materials are promising candidates for gas-sensing applications [29,30].

According to Chung *et al*. [31] and Lee *et al*. [32], WO_3_-based gas sensors with a large surface area have shown a great sensitivity towards NO_2_ gas. Similar to this, Stankova *et al*. [33] have reported WO_3_ thin film deposition by RF sputtering, and the gas response noted was about 5 at 1 ppm of NO_2_ gas at 370°C of operating temperature. While An *et al*. [34] found that hydrothermally synthesized one-dimensional WO_3_ nanorods exhibited good gas-sensing response at 300°C.

An efficient ethanol gas sensor was developed by Liu *et al*. [35] by using WO_3_ · H_2_O nanorods and spherical networks that were synthesized by a simple hydrothermal process. It has been shown that the spherical network has superior gas sensitivity in comparison with the evenly spread nanorods. This may be due to the porous structure being more effective than the uniformly distributed WO_3_ nanorods, and the greatest sensing response was observed for 100 ppm ethanol at 350°C. Zeng *et al*. [36] synthesized WO_3_ · H_2_O with diverse morphologies using a hydrothermal process with various number of surfactants. The optimized composition showed great sensing response to ethanol at 350°C for 400 ppm. WO_3_ and WO_3_ · nH_2_O-based gas sensors worked well when operated at high temperatures, as reported earlier. Due to this, an attempt was made throughout this study to develop a WO_3_ · H_2_O-based gas sensor that could function at temperatures lower than their usual operating temperature. The effectiveness of gas sensing is also discussed in relation to the morphological changes that happened in WO_3_ · H_2_O as a result of variations in hydrothermal temperature.

## 2. Experimental

### 2.1. Chemical

Sodium tungstate dihydrate (Na_2_WO_4_ · 2H_2_O, 99.5%), potassium sulfate (K_2_SO_4_, 98.5%), oxalic acid (H_2_C_2_O_4_, 99%), citric acid (C_6_H_8_O_7_ · H_2_O, 99%), hydrochloric acid (HCl, 36%) and absolute ethanol (C_2_H_5_OH, 99.7%) were used in synthesis process and are of analytical grade. Throughout the synthesis process, deionized (DI) water was used.

### 2.2. Synthesis and fabrication of flower-like tungsten oxide nanostructures

In this synthesis method, 5 mmol Na_2_WO_4_ · 2H_2_O was mixed in 120 ml DI water with constant stirring so as to make a transparent solution, and then a 3 M HCl solution was added drop-wise into the transparent solution until the pH value reached 1. Following this step, a combination of 5 mM K_2_SO_4_ and 5 mM H_2_C_2_O_4_ was added to the previously mentioned mixture. Following that, the mixture was transferred to 200 ml Teflon-lined stainless-steel autoclave and held at 120°C, 160°C and 200°C for 12 h. After gradually cooling to room temperature, the supernatant was taken and rinsed several times with DI water and ethanol before drying for 12 h in an electric oven at 60°C. The synthesized samples are labelled as W1, W2 and W3.

### 2.3. Characterization

X-ray powder diffraction patterns produced using Bruker D8 Advance X-ray diffractometer with bandpass filter Cu K*α* radiation (*λ* = 1.54 Å) in the 2*θ* = 20°−80° range are used to evaluate the crystalline phase of the synthesized samples. Material's optical spectra were measured with a UV–Vis absorption spectroscopy spectrometer (Jasco V750). To analyse the morphology of the synthesized WO_3_ nanomaterial, field-emission scanning electron microscopy (FESEM) was carried out using a FET Nova Nano SEM 450. Fourier transform infrared (FTIR) spectroscopy measurements (Shimadzu FTIR-8900) were taken in the 280–4000 cm^−1^ range. A semiconductor parameter analyser system (Keithley 4200A) was used to measure current–voltage (I–V) characteristics.

### 2.4. Gas sensors fabrication and sensing measurement

Initially, suitable amount of the as-prepared WO_3_ nH_2_O was combined with an organic to make thick slurry and then was screen printed onto the alumina substrate (sensor dimensions = 1 × 1 cm) and dried under IR lamp. Later the prepared sensors were sintered at 400°C to eliminate organic residues and stabilize the sensing signals. The sensor's sensing characteristics were evaluated using a table-top static gas-sensing apparatus, model no. TPD-BARC-16CH [37]. While air and target gas went through the sensor chamber, the sensor's steady resistance in air was found to be *R*_air_ and the sensor's steady resistance in the air–gas combination was determined to be *R*_gas_. The response of the NO_2_ sensor was determined using the *R*_gas_/*R*_air_ ratio.

## 3. Results and discussion

### 3.1. X-ray diffraction studies

**Figure 1.**
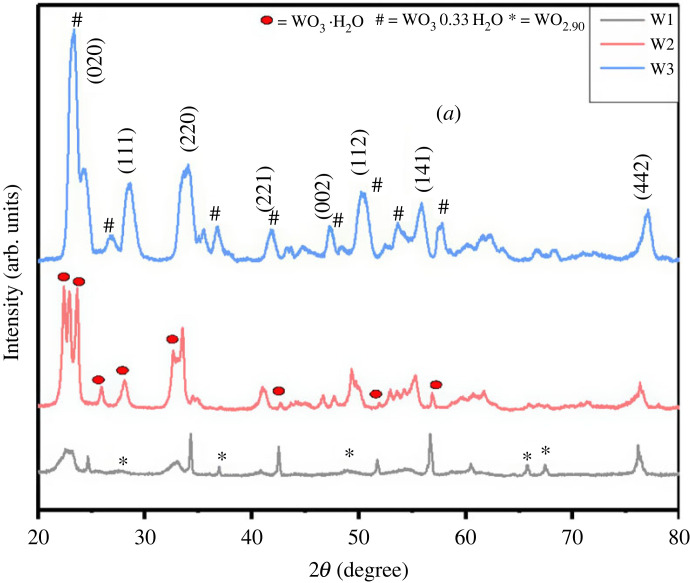
XRD pattern of WO_3_ · nH_2_O synthesis at various hydrothermal temperatures.

X-ray diffraction (XRD) patterns were used to investigate the phase purity and crystallographic structure of nanomaterial prepared at various hydrothermal temperatures. XRD patterns of synthesized WO_3_ · nH_2_O materials obtained at various hydrothermal temperatures are shown in figure 1. The products obtained at 120°C (W1) exhibit a combined phase of hydrated tungsten oxide WO_3_ · H_2_O (JCPDS 36-1142) and WO_2.90_ (*) (JCPDS 73-2182). Materials obtained at 160°C (W2) and 200°C (W3) agree well with standard values of monoclinic WO_3_ · H_2_O (JCPDS 84-0886) with lattice parameter *a* = 5.24, *b* = 1­0.71, *c* = 5.13 and orthorhombic WO_3_ · 0.33H_2_O (#) (JCPDS 72-0199) with lattice parameter *a* = 7.35 *b* = 12.51, *c* = 7.70, respectively. Further, it was also noted that as the hydrothermal temperature was increased, the intensity of the diffraction peaks and crystallinity of the material were also increased, indicating the improvement in the crystallinity of hydrated WO_3_. This is most likely due to the fact that the increased dissolution rate in hydrothermal system at elevated temperature favours the crystal development of WO_3_ · nH_2_O microstructures.

### 3.2. Raman scattering spectroscopy

The degree of tungsten oxide hydration and the molecular vibration of the samples can both be determined using Raman spectroscopy. This information is helpful to explain the structural changes that take place in the material at various synthesis temperatures. The Raman spectra of W1, W2 and W3 nanostructures are shown in figure 5*a*. The existence of a band at about 946 and 636 cm^−1^ in the Raman spectra for the all the samples reflects the primary characteristics that are typical of hydrated WO_3_ [38]. The peak at 660 cm^−1^, which is strongly impacted by hydration, is representative of levels of hydration present in the samples, which is also confirmed from XRD analysis. When the synthesis temperature is between 160°C (W2) and 200°C (W3), the spectrum exhibits four well-defined bands with centres at 272, 325, 716 and 802 cm^−1^. These four bands are somewhat similar to the WO_3_ basic bands. The bending vibrations W-O-W and stretching vibrations O-W-O are often ascribed to the wavenumber ranges of 200–400 and 700–803 cm^−1^, respectively. Further, the lattice modes are responsible for the peak at 180 cm^−1^ [39].

### 3.3. Morphological analysis

**Figure 2.**
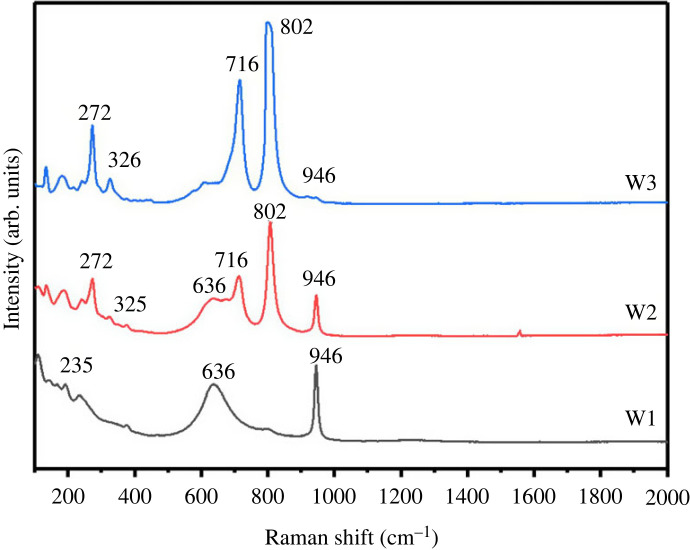
Raman spectra of WO_3_ · n H_2_O synthesis at various temperatures.

**Figure 3.**
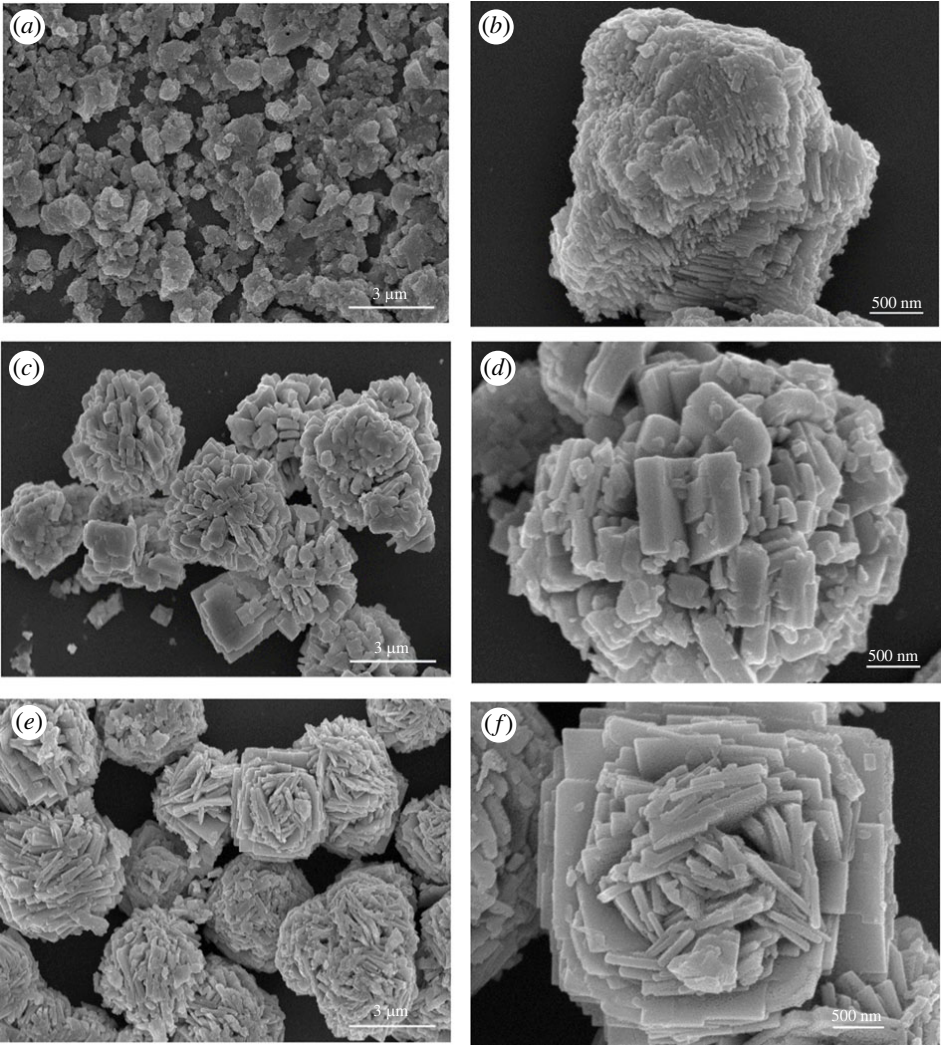
FESEM image of WO_3_ · nH_2_O nanostructures synthesis at various hydrothermal temperatures (*a*,*b*) W1; (*c*,*d*) W2; (*e*,*f*) W3.

The morphology of hydrated WO_3_ powder formed at various temperatures is depicted in figure 2. It can be seen that the hydrothermal synthesis parameters have significant influence on the morphology, particle size, as well as agglomeration of the nanostructures. The WO_3_ · H_2_O powder synthesized at 120°C (W1) formed by stacking of small sheet is observed and is around 50–80 nm in length, as seen from figure 2*a*. When the temperature is elevated from 120°C to 160°C (W2), the WO_3_ · H_2_O sheets grew longer (0.7–1.2 µm in length) and arrange in flower-like pattern. Further, WO_3_ synthesized at 200°C (W3) exhibited a more consistent flower-like structure than W2, resulting in a greater surface area of the partially hydrated WO_3_ powder. This demonstrates that high temperatures provide more energy for hydrothermal processes and promote hydrated WO_3_ crystal formation. As a result, particle size also increases figure 3.

### 3.4. Growth mechanism

**Figure 4.**
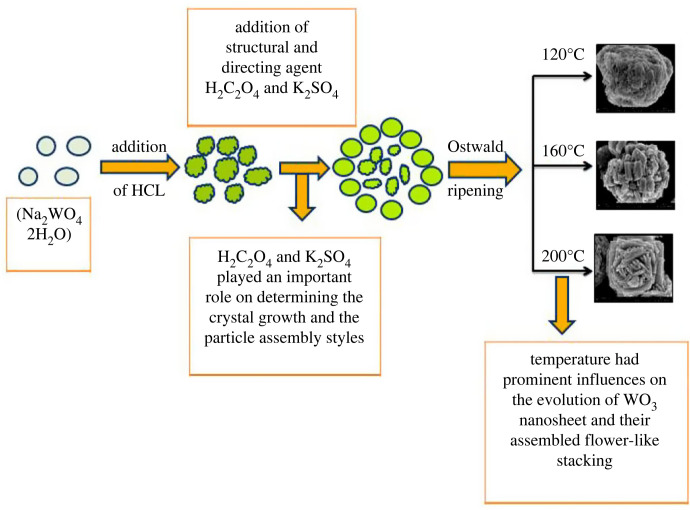
Plausible growth mechanism of WO_3_ · nH_2_O nanostructures.

It is important to study a plausible growth mechanism might be responsible for the development of the uniform WO_3_ · nH_2_O nanosheet staked in flower-like pattern shown in figure 4, The reaction steps may be evaluated as shown below:

3.1 $${\mathrm{Na}}_{2}{\mathrm{WO}}_{4}+2\mathrm{HCl}+{\mathrm{nH}}_{2}O\to H_{2}{\mathrm{WO}}_{4}.nH_{2}O+2\mathrm{NaCl},$$

3.2 $$H_{2}{\mathrm{WO}}_{4}\cdot {\mathrm{nH}}_{2}O\to H_{2}{\mathrm{WO}}_{4}+{\mathrm{nH}}_{2}O,$$

3.3 $$H_{2}{\mathrm{WO}}_{4}\to {\mathrm{WO}}_{3}\cdot {\mathrm{nH}}_{2}O$$

3.4 $$\;\mathrm{and}\;{\mathrm{WO}}_{3}\cdot H_{2}O\to {\mathrm{WO}}_{3}\cdot 0.33H_{2}O+0.67H_{2}O.$$

To reveal the growth mechanism of WO_3_ · nH_2_O, a series of experiments with varying reaction temperatures were carried out while the other reaction parameters remain constant. The morphology of WO_3_ microstructures is greatly influenced by the hydrothermal temperature. Low hydrothermal temperatures result in uneven and aggregated nanostructures of varying sizes; however, increase in the hydrothermal temperature has significant effect and is favourable environment for the formation of nanosheets. This could be related to the effect of K_2_SO_4_ and H_2_C_2_O_4_ as structurally guiding agents. Oxalic acid can control the rate of growth of different faces of WO_3_ by adsorbing on the surfaces of nanoparticles in different ways. It can also act as a selective adsorbent or capping agent. In the case of K_2_SO_4_, the ${\mathrm{SO}}_{4}^{2-}$ ion can only restrict nucleus growth in two directions, resulting in the formation of a one-dimensional crystal. Furthermore, the quantity of K_2_SO_4_ in the particles has a considerable influence on their size. When oxalic acid is used as an additive, two directions of WO_3_ · nH_2_O growth are suppressed, resulting in the formation of miniature flower-like hierarchical structures. The presence of ${\mathrm{SO}}_{4}^{2-}$ ions facilitates the formation of elongated crystallites. [40,41]. In our experiment, firstly Na_2_WO_4_ and HCl interacted to produce H_2_WO_4_ (equation (3.1)) [42]. Second, nucleation began and the WO_3_ · H_2_O crystal nucleus was developed at the initiation of the hydrothermal process. The induction action of Na^+^ and K^+^ then resulted in a large number of WO_3_ · H_2_O primary nanoparticles. When the hydrothermal reaction was carried out for 120°C, nanosheet-based aggregates with round shape were observed. Later, the formation of WO_3_ · H_2_O takes with the formation of WO_2.90_. At 160°C, only WO_3_ · H_2_O was formed with no impurities of WO_2.90_, and sheets staked in flower-like pattern were observed. When the reaction temperature was extended to 200°C, larger sheets anchored in a flower-like pattern were formed more consistently. During the Ostwald ripening, WO_3_ · H_2_O changed into WO_3_ · 0.33H_2_O.

### 3.5. UV–visible analysis

**Figure 5.**
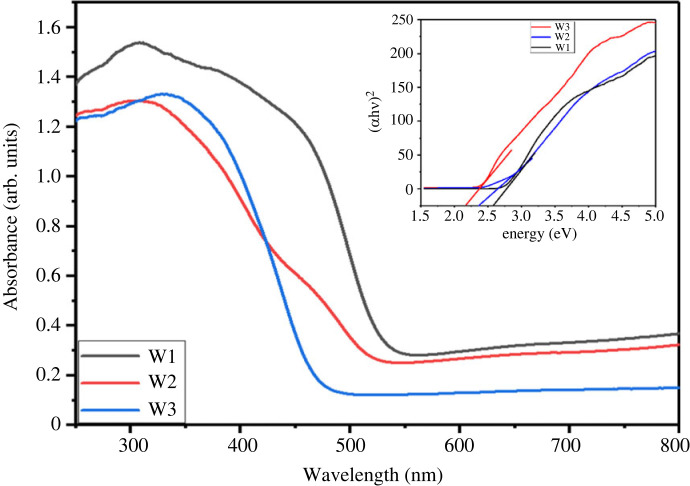
UV–visible absorption spectra of WO_3_ · nH_2_O synthesis at various hydrothermal temperatures.

UV–Vis spectroscopy is used to examine the optical characteristics of hydrated WO_3_ nanostructures, and the results are depicted in figure 5. The threshold values of W2 and W3 were red-shifted in contrast with W1. This red shift in threshold values promotes free carrier formation and might be beneficial for enhancing gas-sensing performance. The band gap energy was calculated from K–M model [43] by plotting a graph of (*αhʋ*)^2^ versus photon energy (*hʋ*) (Tauc plot). The estimated band gap for W1, W2 and W3 was 2.6, 2.4 and 2.2 eV, respectively. Increases in absorbance and particle size suggest a lowering in the band gap of the samples with reaction temperature [44].

### 3.6. Fourier transform infrared spectroscopy analysis

**Figure 6.**
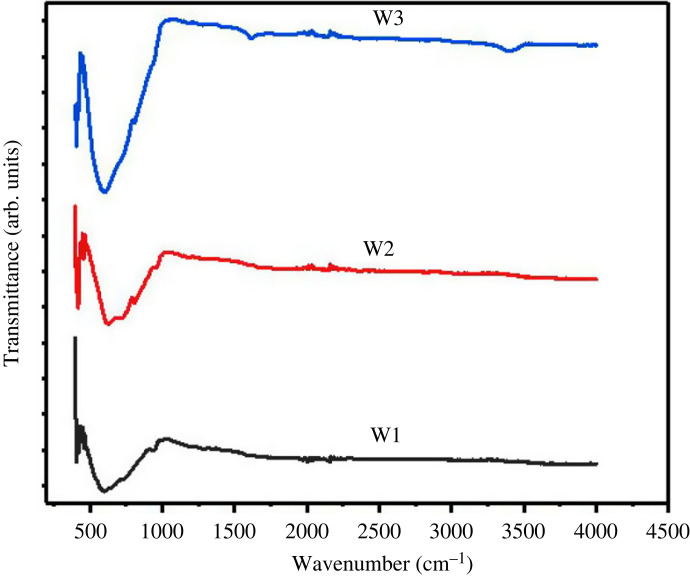
FTIR spectra of synthesized WO_3_ · nH_2_O nanostructures.

The chemical bonding of WO_3_ · nH_2_O microstructure was analysed through FTIR spectroscopy. The FTIR spectra of all the synthesized materials are depicted in figure 6.

A broad absorption band in the wavenumber range of 420–1000 cm^−1^ confirms the synthesis of tungsten oxide due to the vibration modes of the W-O or W=O bond. The FTIR spectra clearly show that the typical absorption bands of tungsten oxide are noticeable for the nanostructure obtained at 200°C (W3). Hence, it can be concluded that higher reaction temperatures tend to form the essential W-O chemical bonding due to the proper growth environment.

### 3.7. Current–voltage characteristics measurement

**Figure 7.**
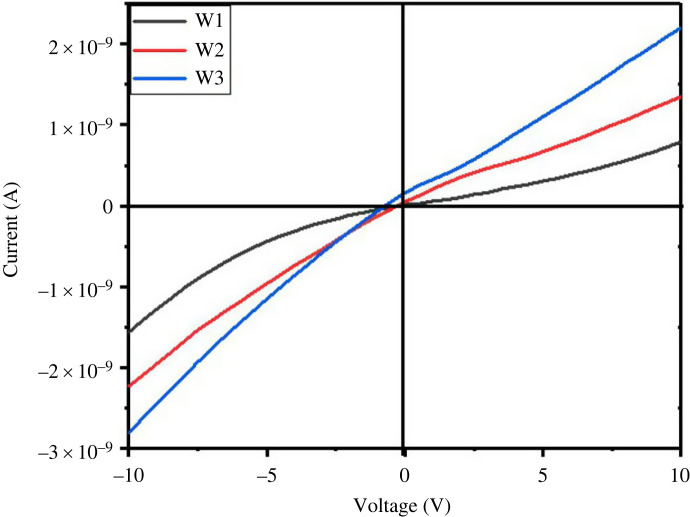
The current–voltage characteristics of the synthesized WO_3_ · nH_2_O nanostructures.

Current–voltage characteristics of WO_3_ · nH_2_O sensors synthesized at various hydrothermal temperatures are shown in figure 7. When a semiconductor and metal come into contact, a Schottky barrier is formed, which results in rectifying (i.e. nonlinear I–V) connections, which are typically undesirable [45]. Synthesized WO_3_ sensors demonstrate linear I–V characteristics with ohmic behaviour for all the synthesized nanostructure sensors. Furthermore, sensors fabricated using W3 nanostructures indicating more connectivity of the grains.

### 3.8. Gas-sensing properties

**Figure 8.**
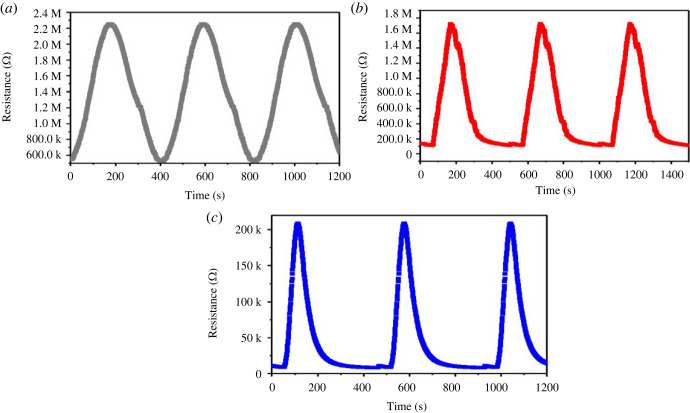
Sensor response of the WO_3_ · nH_2_O (W1 (*a*), W2 (*b*) and W3 (*c*)) sensors at the operating temperature of 185°C.

To study the gas-sensing measurement of synthesized hydrated WO_3_ nanostructures, initially, the operating temperature was optimized, as operating temperature is one of the most essential parameters that determine the precise kinetics that takes place among both NO_2_ and sensitive materials. The sensor response of the fabricated sensors was examined at the optimized operating temperature of 185°C. The behaviour of these gas sensors was plotted and its performance was calculated, as shown in figure 8. Figure 8 shows a remarkable change in resistance when the NO_2_ gas was exposed to all the fabricated sensors (W1, W2 and W3) over the time.

The sensor response increases as the hydrothermal temperature rises. The effect of hydrothermal temperature on detecting ability may be linked to its influence on the morphology and structure of the materials. Hydrothermal temperature modifies morphology of the material, influences its form and defines its unique surface area. As a result of this, large number of active sites was available for NO_2_ adsorption and enhances gas molecule flow in the sensitive layer. Therefore, W3 sensor demonstrated improved NO_2_ (25 ppm) sensing response at 185°C compared with other fabricated sensors (W1 and W2). The resistance-time curves of W1, W2 and W3 sensors toward 25 ppm NO_2_ gas are shown in figure 8, and the sensor response of 3.97, 10.89 and 17.48 was observed for W1, W2 and W3 sensors, respectively.

**Figure 9.**
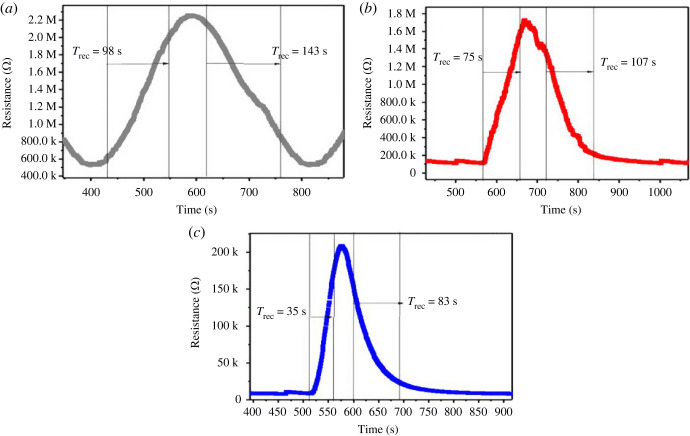
Response–recovery graphs of WO_3_ · nH_2_O sensors at operating temperature of 185°C for 25 ppm of NO_2_ (*a*) W1, (*b*) W2 and (*c*) W2.

The response and recovery characteristics of gas sensors are also essential parameters. In practical applications, particularly real-time monitoring, sensors must have good response and recovery properties. Response and recovery times are known as the time taken by the sensor to accomplish 90% of entire electrical resistance change in the case of adsorption and desorption, respectively. The response/recovery times of the W1, W2 and W3 sensors towards 25 ppm NO_2_ gas are 98 s/143 s, 75 s/107 s and 35 s/83 s, respectively (figure 9). These obtained results indicate that W3 sensor is considerably faster than those of the W1 and W2 sensors.

**Figure 10.**
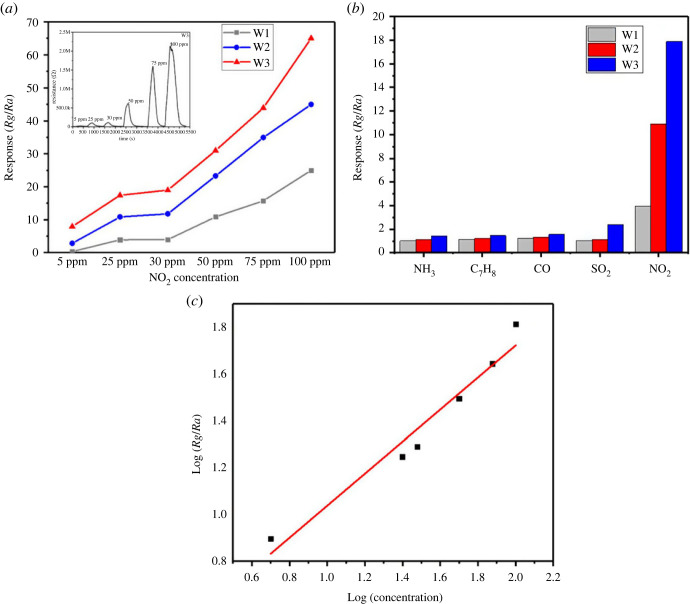
(*a*) Response and recovery curve of WO_3_ · nH_2_O sensor towards NO_2_ with concentrations ranging from 5 to 100 ppm. (*b*) Selectivity measurement of WO_3_ · nH_2_O sensor towards various gases. (*c*) Calibration curve of W3 sensor.

The huge surface area of the sheets staked in a flower-like pattern supplied enough active sites, resulting in a quick gas–surface interaction. However, once the sensor was exposed to the air environment again, the resistance progressively dropped (83 s) to a near-baseline resistance. It is well recognized that fabricating a sensor that is capable of sensing gas at small amounts and allowing gas measurement across a large content is of practical interest. Hence, W1, W2 and W3 sensors were evaluated in 5–100 ppm NO_2_ gas at optimum operating temperature of 185°C to study their sensing properties. The response–recovery curves of W1, W2 and W3 sensors to varied NO_2_ gas concentrations (5–100 ppm) are shown in figure 10*a*. It can be seen that W3 sensor has shown best sensitivity as compared with other sensors. When exposed to 5, 25, 30, 50, 75 and 100 ppm NO_2_ gas, the corresponding response values of the sensor are 7.98, 17.89, 18.21, 31.45, 40.55 and 66.22, which are much larger than those of the W1 and W2 sensors.

Selectivity is another important property for gas sensors in practical implementations. Figure 10*b* depicts the sensor's reactions to several testing gases such as SO_2_, NH_3_, ethanol and NO_2_.

The obtained results show that the response of the W1, W2 and W3 sensor to NO_2_ is greater than that of the other gases, demonstrating that the sensor has a good selectivity for NO_2_. The reason behind the smaller response might be explained as, at given temperature, the adsorbed oxygen on the WO_3_ surface and gases such as NH_3_ and CO could not interact strongly, hence the sensors had a limited response for these gases. Moreover, because NO_2_ has a great affinity for electrons, it may acquire electrons directly from the conduction band as well as react with adsorbed oxygen to obtain electrons at a given temperature. As a result of the selective adsorption and electron exchange of NO_2_ on the WO_3_ · nH_2_O surfaces, considerably good selectivity was achieved. From figure 10*c*, the sensor response exhibits a linear behaviour as a function of the NO_2_ concentration in the log–log plot with the detection limit to 91 ppb.

The effectiveness of WO_3_ and WO_3_ · H_2_O-based sensors for the detection of various gases is summarized in table 1. According to the findings of a prior research, it was observed that our fabricated sensor in this work operates at a lower temperature than that of the reported literatures. Also, our sensors have a lower detection limit, higher response and quicker response and recovery time than other WO_3_ · H_2_O-based sensors. The novelty of this work lies in the fact that to the best of our knowledge WO_3_ · H_2_O sensors have not before been used to detect NO_2_.

**Table 1.** Sensing characteristics of our WO_3_-based gas sensor compared with previous published work.

	gas	operating temperature	concentration	*T*_res_ (s)	*T*_rec_ (s)	response (*R*_g_/*R*_a_)	references
WO_3_ nanorods	NO_2_	300°C	10 ppm	24	300	65	[46]
WO_3_ · nH_2_O nanostructure	ethanol	350°C	100 ppm	2	—		[47]
WO_3_ · H_2_O nanoplate	ethanol	350°C	35 ppm	150	150	25	[48]
Au-loaded WO_3_ · H_2_O	xylene	255°C	5 ppm	1	1	26.4	[49]
WO_3_ nanoflower	NO_2_	200°C	100 ppm	86	150	2.25	[50]
flower-like WO_3_ · 0.33H_2_O	NO_2_	185°C	25 ppm	35	83	17.89	this work

As a result, this procedure is simple to carry out and easily accessible. With all of these positive attributes, we may conclude that our sensor performance is significantly better than the other mentioned in the literature.

### 3.9. Gas-sensing mechanism

The sensing abilities of resistive-type metal oxide gas sensors are strongly determined by the change in resistance of the sensing material as a result of gas contact, where resistance varies according to analyte type and sensor material characteristics. The contact of gas with the surface of flower-like WO_3_ · nH_2_O involved two phenomena, namely adsorption and desorption of atmospheric oxygen molecules on the surface, followed by electrostatic interaction between oxygen species and sensing materials [51,52]. The proposed gas-sensing mechanism is described using schematic diagram as shown in figure 10. When the gas sensor reacts with oxygen, oxygen molecules pick up electrons from the material surface and become charged, resulting in adsorbed oxygen species ($O_{2}^{-}$, O^−^ and O^2−^). Based on the operating temperature, charged oxygen occurs mostly as $O_{2}^{-}$ below 100°C or O^−^ within 100 and 300°C, which is the typical working temperature range. O^2−^ ions are released at 300°C and are then directly assimilated into the lattice.

3.5 $$O_{2}\to O_{2(\mathrm{ads})},\;$$

3.6 $$O_{2(\mathrm{ads}})+e^{-}\to O_{2\left(\mathrm{ads}\right)}^{-},$$

3.7 $$O_{2\left(\mathrm{ads}\right)}^{-}+e^{-}\to 2O^{-}$$

3.8 $$\;\mathrm{and}\;O+e^{-}\to O^{-}.$$

**Figure 11.**
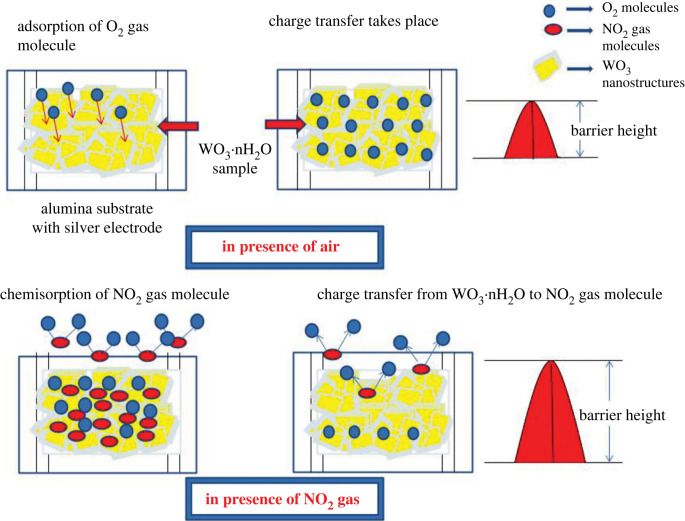
Gas-sensing mechanism.

Once enough oxygen molecules have been absorbed, charge depletion layer forms on the material grains. It is possible to produce steady-state surface oxygen content in air by giving a simple electrical resistance. When the sensor is exposed to NO_2_, it interacts with the adsorbed oxygen species on the surface of the sensing film, trapping electrons from the conduction band and lowering the electron density. It results in the widening of the potential barrier, as seen in figure 11. The following equation represents the sensor surface. Due to the strong electron affinity of compared with the oxygen molecule, the interaction between the gas molecule and adsorbed oxygen species enhanced, resulting in an increase in sensor resistance.

3.9 $${\mathrm{NO}}_{2\left(\mathrm{gas}\right)}+e^{-}\to {\mathrm{NO}}_{2\left(\mathrm{ads}\right)}^{-}$$

and

3.10 $${\mathrm{NO}}_{2(\mathrm{gas})}+O^{-}+2e^{-}\to {\mathrm{NO}}_{2(\mathrm{ads})}^{=}+O_{\left(\mathrm{ads}\right)}^{2-}.$$

The optimized temperature of the WO_3_ sensor in the current work is 185°C; therefore, adsorbed oxygen might be present in O^−^ form. When the flow of NO_2_ gas is terminated, NO_2__(ads)_ ions are desorbed. As a result, the initial situation is re-established. The cycle reactions continue, and therefore NO_2_ detection is accomplished.

## 4. Conclusion

In the present work, WO_3_ · nH_2_O microstructures were successfully synthesized by using hydrothermal technique at various reaction temperatures (120–200°C). The materials’ XRD patterns indicated the monoclinic WO_3_ · H_2_O crystal phase transforms into orthorhombic WO_3_ · 0.33H_2_O as the synthesis temperature increased and the morphological studies showed nanosheet agglomerated to form flower-like structures. Gas-sensing measurements showed that nanostructures synthesized at 200°C (W3) showed higher sensor response of 17.89 towards 25 ppm NO_2_ at 185°C.

## Data Availability

Our data are deposited at the Dryad Digital Repository: https://doi.org/10.5061/dryad.f1vhhmh14 [53].
